# Activation of Leukopoiesis in Rat Blood with Trimecaine-Based Ionic Compounds

**DOI:** 10.1155/2020/7636290

**Published:** 2020-01-13

**Authors:** Lyailya Baktybayeva, Valentina Yu, Alexey Zazybin, Darya Zolotareva, Anuar Dauletbakov

**Affiliations:** ^1^Biology and Biotechnology Department, Al-Farabi Kazakh National University, 050040 Almaty, Kazakhstan; ^2^A. B. Bekturov Institute of Chemical Sciences, 050000 Almaty, Kazakhstan; ^3^Center of Chemical Engineering, Kazakh-British Technical University, 050000 Almaty, Kazakhstan; ^4^School of Chemical & Biochemical Engineering, Satbayev University, 050013 Almaty, Kazakhstan

## Abstract

A study of myelostimulating activity of ionic compounds-trimecaine alkyl iodide derivatives under the cipher BIV (BIV-117, BIV-118, and BIV-119) was conducted on a model of doxorubicin pancytopenia in white laboratory rats. It was established that the compounds BIV-117 and BIV-119 had a pronounced leukopoiesis-stimulating activity, exceeding the activity of the methyluracil as a comparison drug. Compounds BIV-117 and BIV-119 had erythropoiesis- and thrombocytopoiesis-stimulating activity at the level of methyluracil.

## 1. Introduction

Synthetic erythropoietic drugs EPO, Epogen (epoetin alfa), Procrit (r-HuEPO), Mircera (methoxyethylene glycol-epoetin beta), and Aranesp (darbepoietin alfa) are used to stimulate red blood cell (RBC) production in the bone marrow, thereby correcting anemia, minimizing the need for transfusion requirements, and improving the quality of life for patients [1]. Synthetic leukopoiesis-stimulating drugs are divided into low molecular weight and high molecular weight compounds. Low molecular weight synthetic compounds include levamisole ((S)-2,3,5,6-tetrahydro-5-phenylimidazo [2,1-b] thiazole), dibazole (2- (phenylmethyl)-1H-benzimidazole), methyluracil (dioxymethyltetrahydropyrimidine), pentoxyl (5-hydroxymethyl-4-methyluracil), diuciphone (diaminodiphenylsulfone with methyluracil), galavit (phthalhydroside derivative), glutoxim (Bis-(*γ*-L-glutamyl)-L-cysteine-bis-glycine-disodium salt), and others [2]. High molecular weight compounds include polyoxidonium (a derivative of polyethylene piperazine). The most widely used in clinical practice for various forms of myelodepression is the drug methyluracil. Methyluracil is involved in the processes of protein synthesis, stimulating the biosynthesis of nucleic acids (substrate activation). By improving metabolic processes, they accelerate the reproduction and growth of cells, restore the mass and function of damaged organs and tissues, activate leukopoiesis, and increase leukocyte activity. Acting on B-lymphocytes, pyrimidines increase the activity of Krebs cycle enzymes, stimulate their conversion into plasma cells producing immunoglobulins, promote the formation of antibodies, lysozyme, complement, properdine, and interferon, increase the absorption activity of neutrophils and macrophages, affect the body's immune activity, and have anti-inflammatory act [3].

## 2. Materials and Methods

### 2.1. Test Compounds

The compounds BIV-117, BIV-118, and BIV-119 (Table 1) were synthesized in the laboratory of “Ionic Liquids” of the Kazakh-British Technical University [4–7].

### 2.2. Peripheral Blood Hemogram Studies

Myelostimulatory activity of the compounds was performed on a model of doxorubicin pancytopenia. The model was chosen in view of the priority use of myelopoiesis-stimulating drugs in cancer patients after chemotherapy and radiation therapy.

For the studies, 48 adult albino laboratory female rats, 10–15 weeks of age, 210–280 g weight, were used. The animals were obtained simultaneously from one nursery, the biological clinic of the Department of Biology and Biotechnology of the Al-Farabi Kazakh National University. Investigations were carried out in accordance with the Ethical principles and guidelines for scientific experiments on animals [8–10]. All the animals were kept under uniform conditions (wood litter with sawdust, the room temperature 22–24°C, the natural lighting from windows); they were fed a standard feed ration (Delta Feeds Food for laboratory rats and mice full-granulated adaptogenic granular (SPF), article C-19, 2500 kcal/kg, protein content 19% [11]).

Animals were divided into 6 groups of 8 animals. The 6th group of animals was intact. On the 1st, 3rd, and 5th day of the experiment, the 1st, 2nd, 3rd, 4th, and 5th groups of animals were injected intraperitoneally with a 1% solution of doxorubicin hydrochloride [12] at a dose of 10 mg/kg (solvent physiological solution (0.9% aqueous solution of sodium chloride (NaCl))). On the 8th, 10th, and 12th day of the experiment, the animals were intramuscularly injected: the 1st, 2nd, and 3rd groups, 1% solutions of the compounds BIV-117, BIV-118, BIV-119, respectively, at a dose of 5 mg/kg (solvent, physiological saline (0.9% aqueous solution of sodium chloride (NaCl))). The 4th group of placebo was injected intramuscularly with physiological saline (0.9% aqueous solution of sodium chloride (NaCl)), 0.25 ml. The 5^th^ control group was intramuscularly injected with a 1% solution of methyluracil at a dose of 5 mg/kg (solvent physiological solution (0.9% aqueous solution of sodium chloride (NaCl))) (Table 2).

Blood sampling was performed at 09.00 am from the orbital vein of rats in tubes VF-052SDK with 2 mL of EDTA (K2) on the 19^th^ day of the experiment (7 days after injection of BIV-117, BIV-118, and BIV-119) under the mild anesthesia with ether. Blood tests were carried out on a hematology analyzer for animal blood «Abacus junior VET» (Diatron, Denmark). The device is configured to determine the following categories of cells: WBC: total leukocyte count, LYM: absolute lymphocyte count; MID: absolute monocyte-eosinophil count; GRA: absolutegranulocytic count (ex-eosinophil); LY: comparative lymphocyticindicator; MI: comparative monocyte-eosinophil indicator; GR: comparative granulocytic indicator (ex eosinophil); RBC: total erythrocyte value; HGB: hemoglobin; HCT: hematocrit; MCV: the average volume of erythrocytes; MCH: the average content of hemoglobin; MCHC: the average concentration of hemoglobin in red blood cells; RDWC: the latitude distribution of red blood cells; PLT: total platelets volume; PCT: the volume of trombocryte; MPV: the average volume of platelets; PDWc: the latitude distribution of platelets. For double cytological control and accurate separation of granulocytic and agranulocytic subpopulations of blood leukogram, blood smears were stained by the Giemsa stain method, blood leukogram was counted under a microscope SA3300S immersion (magnification 7·100) 100 cells per each smear sample, and then the relative amount of each type of the cells were converted into the absolute value [13].

According to the Instruction for conducting preclinical trials and (or) studies of biologically active substances in the Republic of Kazakhstan [14], when conducting blood tests, it is necessary to double-monitor blood parameters. A visual examination of blood smears allows you to clarify the performance of blood subpopulations and identify elements that do not appear when conducting automated clinical blood tests (for example, a change in the shape of red blood cells, counting immature granulocytes, the presence of altered blood cancer cells, and so on). In myelodepressive conditions, the visual method is mandatory.

### 2.3. Phenotyping of Lymphocytes by Indirect Immunofluorescence

The separation of the erythrocyte, monocyte-granulocyte, and lymphocytic fractions of blood was carried out with a solution of ficoll-urotraste. Phenotyping of lymphocytes was performed by the conventional method of indirect fluorescence using monoclonal antibodies: ICO 111 (CD3^+^), ICO 101 (CD4^+^), ICO 31 (CD8^+^), and ICO 180 (CD20^+^) (MedBioSpektr, Moscow). Cellular complexes with antibodies were suspended in 20 *μ*l of a working solution of FITC conjugates of secondary antibodies (FITC-anti-mouse, manufactured by MedBioSpektr, Moscow) and incubated for 30 minutes at 4°C in a moist thermostat. Cells were fixed in cell fixation solution (8% formalin solution and 4% paraformaldehyde solution). The cell suspension was placed in a well, carved in a Parafilm strip, and glued to a glass slide. Since the recording of the results of the reaction was carried out on fluorescent microscope, the slides were not covered with cover glasses, and the lens was carefully placed in the well on the glass, using a 50% glycerol solution as the immersion medium. All laboratory procedures were carried out in a darkened room. A 100x microscope objective and a 10x eye piece were used. Luminous cells were counted (ring-shaped glow). The reaction is short-lived, so the results of the reaction should be calculated during 24 hours after the formulation of the reaction, keeping the glass in a dark room. Microscopic examination was carried out in a dark room. Due to the manual immunofluorescence typing of lymphocytes, it was possible to visually fix not only active lymphocytes with attached surface markers but also resting cells.

Statistical processing of the results was carried out with the reduction of the average value, the average error, and the confidence interval of the student.

## 3. Results and Discussion

As a result of intoxication with the cytostatic drug doxorubicin hydrochloride, there was a decrease in leukocyte, erythrocyte, and platelet counts. The following blood parameters were recorded: the total leukocyte index decreased up to 5.11 times on the 9^th^ day of observation. The relative lymphocytic index, relative granulocytic index, the level of monocytes, and eosinophils decreased. The absolute lymphocytic index significantly decreased in 6.88 times. The absolute granulocyte index decreased in 5.85 times. Thus, after the administration of cytostatic doxorubicin hydrochloride, leukopenia was recorded in the blood leukogram against the background of absolute lymphocytopenia and granulocytopenia (Table 3).

Changes were recorded in erythrocyte blood counts. The hemoglobin index rapidly decreased in 1.5 times. The average hemoglobin content in red blood cells and the color indicator also decreased. Total platelet count decreased in 7.94 times (Table 3). Thrombocrite level also decreased.

Intoxication of the organism with cytostatics doxorubicin hydrochloride led to pancytopenia against the background of severe leukopenia, erythropenia, and thrombocytopenia. Leukopenia manifested as granulocytopenia and lymphocytopenia.

On the background of artificially induced pancytopenia, the test compounds of the BIV-117, BIV-118, and BIV-119 series were administered to the laboratory rats with blood sampling on the 7^th^ day after the last injection of the compounds.

BIV-118 did not exceed the comparison drug on methyluracil by leukopoiesis-stimulating activity. All relative and absolute indicators of blood leukograms in the studied groups were slightly inferior to those in the control group (Table 4).

The high erythropoiesis and thrombocytopoiesis stimulating activity of the compound BIV-118 should be noted. The compound BIV-118 stimulated the proliferative activity of the erythropoietic pool in a rather short period of time and restored the erythrocyte index to the level of intact animals. The hemoglobin index in the administration group of the compound BIV-118 did not reach the level of intact animals, but was higher than the value of the control group. The hemoglobin value was higher in the control group in 1.29 times and comparable with the indicator in the intact group (Table 4).

BIV-118 effectively stimulated thrombocytopoiesis in the rat. The indicator of the experimental group exceeded that of the control group and was at the level of intact animals (Table 4). The thrombocrit index was comparable with the general platelet index and was high. The following pattern has been noticed long ago: if the compound successfully stimulates the proliferation of the erythrocyte pool, then it will effectively stimulate the thrombocytopoiesis pool. This pattern was confirmed in studies with the compound BIV-118. The compound equally effectively stimulated erythro- and thrombocytopoiesis.

The compounds BIV-117 and BIV-119 effectively stimulated leukopoiesis and the recovery of leukocyte populations was more pronounced in lymphocytic subpopulations. The total leukocyte index in the group of administration of the compound BIV-119 was higher than that in the control group in 2.09 times. The absolute lymphocytic index versus the control group indicator exceeded 2.67 times. The relative lymphocytic indicator of the leukogram of the blood of animals confirmed the high absolute lymphocytic index. Consequently, despite the fact that the relative lymphocyte index was markedly high, but it was within the normal range. The relative granulocyte index against the value of the control group and the intact group exceeded 2.79 and 2.56 times, respectively. The absolute granulocyte index in the group with the introduction of the compound BIV-119 comparable with the indicator of the control group and was lower than the indicator of the intact group in 2.90 times (Table 4).

The BIV-117 compound effectively stimulated leukopoiesis, but was inferior to the BIV-119 compound. The overall leukocyte indicator in the group of administration of the compound BIV-117 was higher than that in the control group in 1.72 times and lower than that in intact animals in 1.34 times. The absolute lymphocytic index in this group of administration was higher than that in the control group in 2.20 times and comparable with the indicator of the intact group. The relative lymphocytic index exceeds the control group and the values in the intact group in 1.27 and 1.24 times, respectively. The relative granulocyte index was lower than that in the control group and the intact group in 3.84 and 3.52 times, respectively. The absolute granulocyte index in the group with the introduction of the compound BIV-117 was comparable with the indicator of the control group and was lower than that in the intact groups in 4.53 times (Table 4).

The erythropoiesis- and thrombocytopoiesis-stimulating activity of the compounds BIV-117 and BIV-119 was at the level of the comparison drug methyluracil. The total erythrocyte index in the groups with the introduction of compounds BIV-117 and BIV-119 was with the value in the control group, but lower than that in intact animals. The hemoglobin level was effectively restored and was at the level of the control group, but lower than the value of intact animals in 1.30 and 1.20 times, respectively (Table 4). The thrombocytopoiesis-stimulating activity of the studied compounds was at the level of the comparison drug methyluracil (Table 4).

Since the compounds exhibited lymphopoiesis-stimulating activity at the second stage of the pharmacological screening, the impact of azaheterocyclic compounds on the proliferation of individual subpopulations of CD3^+^–T-lymphocytes, CD20^+^–B-lymphocytes, CD4^+^–T-helper cells, and CD8^+^–T-cytotoxic cells was evaluated.

A relatively high lymphopoiesis-stimulating activity in the series of BIV-117, BIV-118, and BIV-119 compounds was shown. The active compound BIV-119 increased the absolute CD3^+^–T-lymphocytic index correlating with the value of intact animals, reliably exceeding the animals of the placebo group in 7.28 times and the indicator of the control group in 2.69 times. The indicator CD20^+^–B-lymphocytes was close to the value of intact animals exceeding that of the control group in 3.88 times and indicators of the placebo group in 4.69 times. The level of CD4^+^–T-helper cells approached the value of intact animals and was significantly higher than that of the placebo group in 8.91 times and the values of the control group in 1.87 times. The level of CD8^+^–T–cytotoxic cells approached the value of intact animals, but exceeded that of the placebo group in 7.61 times and the control group in 2.91 times. Immunoregulatory indexes in the group of administration of the compound BIV-119 and in the intact group were identical to each other and amounted to 1.08 conventional units.

The compound BIV-117 was inferior in lymphopoiesis-stimulating activity to compound BIV-119 also stimulated lymphopoiesis, but the indices of T- and B-lymphocytes moderately reached the average indices of lymphocytic subpopulations. In the group of administration of the compound BIV-117, the level of CD3^+^–T-lymphocytes reached the value of intact animals, exceeding indicators of the placebo group in 3.7 times and the control group in 1.39 times. The recovery of the CD20^+^–B-lymphocytic index was also moderate. It reached the value which was lower than the values of the intact group and the group with the introduction of the BIV-119 in 1.23 times and 1.06 times, respectively. But CD20^+^–B-lymphocytic value in the group of administration of the BIV-117 was significantly lower than that in the control group in 3.65 times and the placebo group in 4.41 times (Table 5). The ratio of indicators CD4^+^–T-helper cells and CD8^+^–T-cytotoxic cells, i.e., immunoregulatory index in the group of administration of the compound BIV-117 was better than in the group of administration of the compound BIV-119 and was 1.12 conventional units. The level of СD4^+^–T-helper cells in the BIV-117 compound administration group was lower than that in the BIV-119 compound administration group and intact group in 2.32 and 2.73 times, respectively. But, the CD4^+^–T-helper index in the BIV-117 administration group was close to that in the control group and exceeded the value of the placebo group in 3.83 times. A similar level of the indicator was in the subpopulations of CD8^+^–T-cytotoxic cells. Indicator CD8^+^–T–lymphocytes in the BIV-117 compound administration group was lower than those in the BIV-119 compound administration group in 2.41 times and the intact in 2.82 times. But, the CD8^+^–T-cytotoxic cells in the group with the introduction of the compound BIV-117 was higher than those in the control group in 1.20 times and was significantly higher than that in the placebo group in 3.15 times (Table 5).

In the series of the studied compounds, the compound BIV-118 showed a relatively low lymphopoiesis stimulating activity. It was inferior in activity to the compounds BIV-119 and BIV-117 and to methyluracil. The lymphocyte values in the BIV-118 administration group were similar to those of the placebo group. In the group of administration of the compound BIV-118, the level of CD3^+^–T-lymphocytes was lower than the value of the intact group in 6.6 times, lower than that of the control group in 2.44 times. Also, CD3^+^–T-lymphocyte indicator was inferior to the value of the BIV-117 administration group and especially the value of the BIV-119 compound administration group in 3.41 and 6.72 times, respectively. The value of CD20^+^–B–lymphocytes in the group of administration of the compound BIV-118 was lower than that in the intact group and groups with BIV-119 and BIV-117 in 3.9 and 3.36 times, respectively (Table 5). The value of CD20^+^–B-lymphocytes in the group of administration of the compound BIV-118 correlated with the indicator of the group of administration of the drug methyluracil. Indicators of CD4^+^–T-helper and CD8^+^–T-cytotoxic lymphocytes were at the same level, and accordingly, the immunoregulatory index was 1.00 conventional units. The low values of both T-lymphocyte subpopulations of the BIV-118 administration group were lower than the values of all control groups: control, placebo, and intact.

Thus, the compounds BIV-117 and BIV-119 had a leukopoiesis-stimulating activity exceeding the activity of the comparison drug methyluracil. The compound BIV-119 had a pronounced leukopoiesis-stimulating activity and moderate–BIV-117. The recovery of leukocytes proceeded with the prevalence of activation of lymphocytopoiesis. The recovery of absolute indices in CD-lymphocytic subpopulations proceeded without disturbing the immunoregulatory index. Also, the compounds BIV-117 and BIV-119 had erythropoiesis and thrombocytopoiesis-stimulating activity at the level of the comparison drug methyluracil. Compound BIV-118 was lower in leukopose-stimulating activity than in compounds BIV-117 and BIV-119 on a level with methyluracil, while platelet- and erythropoiesis-stimulating activity exceeded the reference drug methyluracil.

## Figures and Tables

**Table 1:** Formulas of the compounds.

Compound	Structure	Name
BIV-117	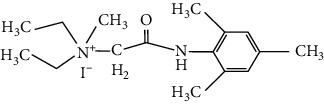	N,N-diethyl-2-(mesitylamino)-N-methyl-2-oxoethanaminium iodide
BIV-118	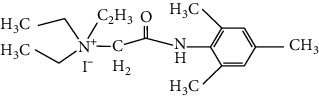	N,N,N-triethyl-2-(mesitylamino)-2-oxoethanaminium iodide
BIV-119	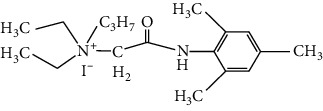	N,N-diethyl-N-(2-(mesitylamino)-2-oxoethyl) propan-1-aminium iodide

**Table 2:** Types of experimental groups.

No	Groups	Administration of doxorubicinhydrochloride	Drug administration
1	Experimental	Yes	BIV-117
2	Experimental	Yes	BIV-118
3	Experimental	Yes	BIV-119
4	Placebo	Yes	Physiological solution
5	Control	Yes	Methyluracil
6	Intact	No	No

**Table 3:** Indicators of blood hemograme with intoxication.

Groups	Intact		After intoxication		
Indicators	Δ*X*	Δ*d*	Δ*X*	Δ*d*	
WBC (10^9^/L)	12.1	0.8	2.37	0.16	*p* ≤ 0.05
LYM (10^9^/L)	7.71	0.1	1.12	0.2	*p* ≤ 0.05
MID (10^9^/L)	0.76	0.01	0.12	0.1	
GRA (10^9^/L)	3.63	0.01	0.62	0.3	*p* ≤ 0.05
LY (%)	63.72	1.1	47.2	1.8	
MI (%)	6.28	0.1	4.9	1.3	
GR (%)	30.0	0.8	26.18	4.5	
RBC (10^12^/L)	7.5	0.9	4.93	0.5	
HGB (g/L)	140.7	8.9	90.75	6.2	
HCT (%)	39.8	1.2	21.21	7.79	
MCV (fl)	55.1	0.1	52.75	1.25	
MCH (pg)	19.0	0.1	17.45	1.15	
MCHC (g/dL)	350.6	14.28	347.25	3	
RDWC	14.0	0.9	13.68	0.5	
PLT (10^9^/L)	660.0	12.2	70.5	23.33	*p* ≤ 0.05
PCT (%)	0.44	0.01	0.05	0.03	
MPV (fl)	7.9	0.1	5.28	2	
PDWC	46.0	0.1	23.1	8.6	

**Table 4:** Indicators of blood hemogram in the comparison group.

Groups	BIV-117		BIV-118		BIV-119		Intact		Control		Placebo		
Indicators	Δ*X*	Δ*d*	Δ*X*	Δ*d*	Δ*X*	Δ*d*	Δ*X*	Δ*d*	Δ*X*	Δ*d*	Δ*X*	Δ*d*	
WBC (10^9^/L)	8.97	0.8	4.8	0.1	10.9	0.7	12.1	0.8	5.2	0.8	3.84	0.93	
LYM (10^9^/L)	7.1	0.4	2.6	0.1	8.6	0.2	7.71	0.1	3.22	0.03	2.22	0.9	$\begin{array}{c} p_{1–5}\leq 0.05,p_{3–5}\leq 0.05 \end{array}$
MID (10^9^/L)	1.1	0.02	0.63	0.01	1.08	0.01	0.76	0.01	0.27	0.001	0.19	0.01	
GRA (10^9^/L)	0.8	0.01	1.52	0.1	1.25	0.01	3.63	0.31	1.7	0.04	1.44	0.13	*p*_1–5_ ≤ 0.05
LY (%)	79.3	0.8	55.1	2.1	78.55	2.1	63.72	1.1	62.04	3.93	57.6	1.65	
MI (%)	12.2	0.8	13.2	1.1	9.75	1.2	6.28	0.1	5.28	0.4	4.84	5.3	
GR (%)	8.5	0.6	31.7	2.1	11.7	0.9	30.0	0.8	32.68	1.6	37.56	9.3	$\begin{array}{c} p_{1–5}\leq 0.05,p_{3–5}\leq 0.05 \end{array}$
RBC (10^12^/L)	5.81	0.5	7.2	0.9	5.02	0.5	7.5	0.9	5.69	0.36	4.67	0.1	$\begin{array}{c} p_{2–5}\leq 0.05,p_{2–6}\leq 0.05 \end{array}$
HGB (g/L)	116.0	12.2	137.0	12.2	107.1	12.1	140.7	8.9	106.0	12.2	96.6	1	$\begin{array}{c} p_{2–5}\leq 0.05,p_{2–6}\leq 0.05 \end{array}$
HCT (%)	30.4	1.2	39.2	2.1	27.4	2.1	39.8	1.2	34.69	0.7	28.1	0.84	
MCV (fl)	52.3	2.2	54.5	2.2	54.5	2.1	55	0.1	52.5	1.5	50	11.3	
MCH (pg)	20	0.8	19	1.2	21.35	1.2	19	0.1	18.45	0.55	17	0.3	
MCHC (g/dL)	382	23.2	349	18.8	391.5	16.5	350.6	14.28	349.25	14.4	342.5	6.5	
RDWC	13.3	0.3	15.0	0.1	16.15	0.1	14	0.9	12.7	0.4	13.55	0.22	
PLT (10^9^/L)	543.0	25.5	639.0	13.8	503.5	23.1	660.0	12.3	518.25	19.9	447	25.1	
PCT (%)	3.8	0.1	4.35	0.1	3.43	0.1	0.44	0.01	0.31	0.06	0.32	0.04	
MPV (fl)	7.2	0.8	6.8	0.8	7	0.5	7.9	0.1	6.63	0.3	7.1	0	
PDWC	15.1	0.5	14.8	0.6	14.9	0.8	46	0.1	30.95	0.25	32.35	1.3	

**Table 5:** Indicators of blood lymphogramme in the comparison group.

No		WBC (10^9^/L)/%	LYM (10^9^/L)/%	CD3^+^ (10^9^/L)/%	CD20^+^ (10^9^/L)/%	CD4^+^ (10^9^/L)/%	CD8^+^ (10^9^/L)/%
1	BIV-117	(8.9 ± 0.8)/100	(7.05 ± 0.4)/(79.3 ± 0.8)	(2.22 ± 0.3)/(31.3 ± 1.2)	(1.90 ± 0.0)/(26.9 ± 7.1)	(0.46 ± 0.0)/(21.0 ± 3.5)	(0.41 ± 0.01)/(5.7 ± 0.04)
2	BIV-118	(4.8 ± 0.1)/100	(2.64 ± 0.1)/(55.1 ± 2.1)	(0.65 ± 0.0)/(25.1 ± 1.1)	(0.61 ± 0.0)/(23.4 ± 0.4)	(0.10 ± 0.0)/(16.5 ± 0.3)	(0.16 ± 0.07)/(6.1 ± 0.39)
3	BIV-119	(10.9 ± 0.7)/100	(8.55 ± 0.2)/(78.5 ± 2.1)	(4.37 ± 0.9)/(50.9 ± 0.7)	(2.02 ± 0.0)/(23.5 ± 0.4)	(1.07 ± 0.0)/(24.5 ± 0.9)	(0.99 ± 0.01)/(11.5 ± 0.41)
4	Intact	(12.1 ± 0.8)/100	(7.70 ± 0.1)/(63.7 ± 1.1)	(4.29 ± 0.6)/(55.7 ± 3.2)	(2.34 ± 0.0)/(30.4 ± 2.4)	(1.26 ± 0.0)/(29.5 ± 1.4)	(1.16 ± 0.04)/(15.0 ± 0.6)
5	Placebo	(3.8 ± 0.9)/100	(2.18 ± 0.9)/(57.6 ± 1.6)	(0.60 ± 0.0)/(27.4 ± 2.1)	(0.43 ± 0.0)/(19.4 ± 0.6)	(0.12 ± 0.0)/(20.4 ± 0.3)	(0.13 ± 0.00)/(5.93 ± 0.81)
6	Сontrol	(5.2 ± 0.8)/100	(3.22 ± 0.3)/(62.0 ± 3.9)	(1.59 ± 0.7)/(49.6 ± 1.6)	(0.52 ± 0.0)/(16.3 ± 2.7)	(0.57 ± 0.0)/(36.4 ± 3.3)	(0.34 ± 0.02)/(10.6 ± 0.60)
7		$\begin{array}{c} p_{1–5}\leq 0.05,p_{1–4}\leq 0.05,p_{2–4}\leq 0.05,p_{3–5}\leq 0.05,p_{3–6}\leq 0.05 \end{array}$	$\begin{array}{c} p_{1–5}\leq 0.05,p_{1–6}\leq 0.05,p_{3–5}\leq 0.05,p_{3–6}\leq 0.05 \end{array}$	$\begin{array}{c} p_{1–5}\leq 0.05,p_{1–6}\leq 0.05,p_{3–5}\leq 0.05,p_{3–6}\leq 0.05 \end{array}$		$\begin{array}{c} p_{1–4}\leq 0.05,p_{2–4}\leq 0.05,p_{3–5}\leq 0.05,\\ p_{3–6}\leq 0.05 \end{array}$	$\begin{array}{c} p_{1–6}\leq 0.05,p_{1–4}\leq 0.05,p_{2–4}\leq 0.05,p_{2–6}\leq 0.05 \end{array}$

## Data Availability

Data used to support the findings of this study are included within the article. Data are available from the corresponding author (Alexey Zazybin; azazybin@yahoo.com) for researchers who meet the criteria for access to confidential data.

## References

[1] Palmer S. C., Saglimbene V., Mavridis D. (2014). Erythropoiesis-stimulating agents for anaemia in adults with chronic kidney disease: a network meta-analysis. *Cochrane Database of Systematic Reviews*.

[2] Werner G. H., Jolles P. (1996). Immunostimulating agents: what next? A review of their present and potential medical applications. *European Journal of Biochemistry*.

[3] Grih V. V., Krasnyuk I. I. (2016). Prospects for the use of solid dispersions of methyluracil in medicine and pharmacy. *Pharmacy*.

[4] Zolotareva D. S., Basharimova A. A., Bayazit S., Yu V. K., Zazybin A. G. (2017). N-ethoxyethylpiperidine, trimecaine and piromecaine based ionic compounds: synthesis and prediction of biological activity. *International Journal of Chemical Engineering and Applications*.

[5] Zazybin A., Yu V., Baktybayeva L., Zolotareva D., Naukanova M. (2019). Myelostimulator.

[6] Zazybin A., Yu V., Anapiyayev B. (2019). Plant growth stimulator.

[7] Yu V., Ten A., Baktybayeva L. (2018). Synthesis and biological evaluation of 1.3.8-Triazaspiro[4.5]decane-2,4-dione derivatives as myelostimulators. *Journal of Chemistry*.

[8] Order of the minister of health of the republic of Kazakhstan, On Approval of Preclinical (Non-clinical) Studies of Biologically Active Substances, No. 745, November 2009

[9] Approval No. 672 of September 18. 2017 of the Local Ethics Committee of the Faculty of Medicine–Higher School of Public Health of the Al-Farabi Kazakh National University (IRB00010790 al-Farabi Kazakh National University IRB)

[10] Permission of the local ethical commission of the Kazakh National Medical University named after S. D. Asfendiyarova No. 7 (58) of September 12. 2017

[11] http://www.biopro-company.tiu.ru

[12] Praliev K. D., Iskakova T. K., Baktybaeva L. K., Malmakova A. E. (2015). Synthesis and myelostimulatory activity of a number of 3.7-diazabicyclo[3.3.1] nonane derivatives. *Pharmaceutical Chemistry Journal*.

[13] Wu A. N. B. (2006). *Tietz Clinical Guide to Laboratory Tests*.

[14] Order of the Minister of Health of the Republic of Kazakhstan Dated February 14. 2005 No. 51

